# In Vivo Molecular K-Edge Imaging of Atherosclerotic Plaque Using Photon-counting CT

**DOI:** 10.1148/radiol.2021203968

**Published:** 2021-05-04

**Authors:** Salim A. Si-Mohamed, Monica Sigovan, Jessica C. Hsu, Valérie Tatard-Leitman, Lara Chalabreysse, Pratap C. Naha, Thibaut Garrivier, Riham Dessouky, Miruna Carnaru, Loic Boussel, David P. Cormode, Philippe C. Douek

**Affiliations:** From the University of Lyon, National Institute of Applied Sciences of Lyon, University Claude Bernard Lyon 1, Jean Monnet University–Saint Etienne, French National Centre for Scientific Research, Institut national de la santé et de la recherche médicale, Centre de Recherche en Acquisition et Traitement de l’Image pour la Santé Unité mixte de recherche 5220, U1206, F‐69621, Lyon, France (S.A.S.M., M.S., V.T.L., R.D., L.B., P.C.D.); Departments of Radiology (S.A.S.M., T.G., L.B., P.C.D.) and Pathology (L.C.), Hospices Civils de Lyon, Lyon, France; Department of Radiology, Hospital of the University of Pennsylvania, Philadelphia, Pa (J.C.H., P.C.N., D.P.C.); Department of Radiology, Faculty of Medicine, Zagazig University, Egypt (R.D.); and Department of Rheumatology, Allergy, and Immunology, Yale University, New Haven, Conn (M.C.).

## Abstract

**Background:**

Macrophage burden is a major factor in the risk of atherosclerotic plaque rupture, and its evaluation remains challenging with molecular noninvasive imaging approaches. Photon-counting CT (PCCT) with k-edge imaging aims to allow for the specific detection of macrophages using gold nanoparticles.

**Purpose:**

To perform k-edge imaging in combination with gold nanoparticles to detect and quantify the macrophage burden within the atherosclerotic aortas of rabbits.

**Materials and Methods:**

Atherosclerotic and control New Zealand white rabbits were imaged before and at several time points up to 2 days after intravenous injection of gold nanoparticles (3.5 mL/kg, 65 mg gold per milliliter). Aortic CT angiography was performed at the end of the follow-up using an intravenous injection of an iodinated contrast material. Gold k-edge and conventional CT images were reconstructed for qualitative and quantitative assessment of the macrophage burden. PCCT imaging results were compared with findings at histologic examination, quantitative histomorphometry, transmission electron microscopy, and quantitative inductively coupled plasma optical emission spectrometry. Pearson correlations between the macrophage area measured in immunostained sections and the concentration of gold and attenuation measured in the corresponding PCCT sections were calculated.

**Results:**

Seven rabbits with atherosclerosis and four control rabbits without atherosclerosis were analyzed. In atherosclerotic rabbits, calcifications were observed along the aortic wall before injection. At 2 days after injection of gold nanoparticles, only gold k-edge images allowed for the distinction of plaque enhancement within calcifications and for lumen enhancement during angiography. A good correlation was observed between the gold concentration measured within the wall and the macrophage area in 35 plaques (five per rabbit) (*r* = 0.82; 95% CI: 0.67, 0.91; *P* < .001), which was higher than that observed on conventional CT images (*r* = 0.41; 95% CI: 0.09, 0.65; *P* = .01). Transmission electron microscopy and inductively coupled plasma optical emission spectrometry analyses confirmed the gold k-edge imaging findings.

**Conclusion:**

Photon-counting CT with gold nanoparticles allowed for the noninvasive evaluation of both molecular and anatomic information in vivo in rabbits with atherosclerotic plaques.

Published under a CC BY 4.0 license.

*Online supplemental material is available for this article.*

See also the editorial by Leiner in this issue.

## Graphical abstract

**Figure:**
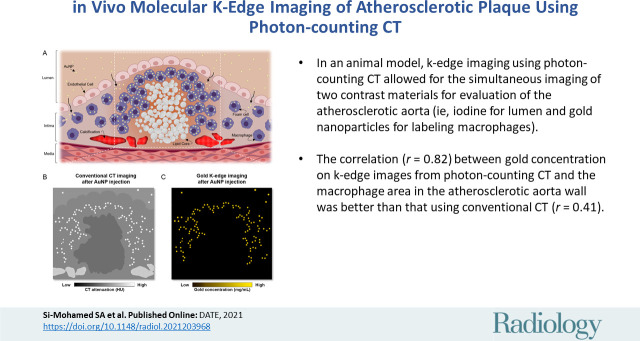

**Summary**

Photon-counting CT k-edge imaging combined with gold nanoparticles allowed for the quantitative molecular imaging of macrophages in atherosclerotic plaques despite calcifications and vascular iodinated enhancement.

**Key Results**

■ In an animal model, k-edge imaging using photon-counting CT allowed for the simultaneous imaging of two contrast materials for evaluation of the atherosclerotic aorta (ie, iodine for lumen and gold nanoparticles for labeling macrophages).
■ The correlation (*r* = 0.82) between gold concentration on k-edge images from photon-counting CT and the macrophage area in the atherosclerotic aorta wall was better than that using conventional CT (*r* = 0.41).

## Introduction

A therosclerosis is a leading cause of coronary artery ­disease worldwide, with a high risk of myocardial infarction as the first major clinical manifestation (1). Noninvasive imaging of the macrophage burden, an important determinant of atherosclerotic plaque vulnerability, can assist in diagnosing patients who are at a high risk of rupture (2,3). CT imaging is a method of choice for coronary artery disease, and it produces thin-section images of coronary arteries in less than 1 second (4). However, current CT technology would be greatly improved by the following: *(a)* better spatial resolution, *(b)* specific imaging capabilities to differentiate iodine contrast material from wall calcifications that have a similar attenuation value, and *(c)* a specific contrast material to target inflammatory processes in plaques. Recently developed photon-counting CT (PCCT) may be useful to address these challenges.

PCCT uses small detectors to enable high-spatial-­resolution imaging (5–8). In addition, these detectors resolve the energy of each photon of the transmitted spectrum, quantify them, and classify them into energy bins. This process permits a configurable sampling of the energy-dependent attenuation of a subject to discriminate between Compton and photoelectric effects that are specific for a given material (6). In the presence of one or more exogenous materials with high atomic numbers (ie, 60 or higher), PCCT enables k-edge imaging to produce quantitative maps of the distribution of an individual element. This is possible because the system is capable of detecting the discontinuity of attenuation around the k edge (5,6). Therefore, a k-edge contrast material that accumulates in macrophages could be extremely advantageous for k-edge imaging of vulnerable atherosclerotic plaques.

Among the candidates for k-edge imaging, promising results have been reported using gold nanoparticles (9,10). In addition, gold nanoparticles are highly biocompatible, are extremely dense, and have low viscosity, which is important for in vivo applications (11). To date, PCCT has shown encouraging results for cardiovascular imaging in phantoms, animals, and human studies (7,12–14), including proof of concept of k-edge imaging for assessing atherosclerotic change (9,15–19).

Therefore, we performed PCCT-enabled k-edge imaging in combination with gold nanoparticles to detect and quantify the macrophage burden within the atherosclerotic aortas of rabbits. K-edge imaging results were compared with analyses of conventional CT imaging, findings at histologic examination, transmission electron microscopy, and quantitative inductively coupled plasma optical emission spectrometry.

## Materials and Methods

### Animal Model of Atherosclerotic Plaque

All procedures were conducted with approval from the institutional animal care and use committee (Comité d’éthique pour l’expérimentation animale neurosciences Lyon, Autorisation de projet utilisant des animaux à des fins scientifiques Ministry of Research no. 1732–2015091411181645) between September 2017 and January 2020. Eleven male New Zealand white rabbits (Charles River Laboratories) were used. Seven of the 11 rabbits were fed a 1% cholesterol diet (Safe Diets) for 2 weeks before inducing a balloon injury of the aorta, as previously described (20,21). The same diet was followed for a further 6 weeks then substituted with a chow diet for 10 weeks. The four uninjured rabbits with matching weight and sex were fed a regular chow diet and used as nonatherosclerotic controls.

### PCCT Imaging Protocol

**Figure 1:**
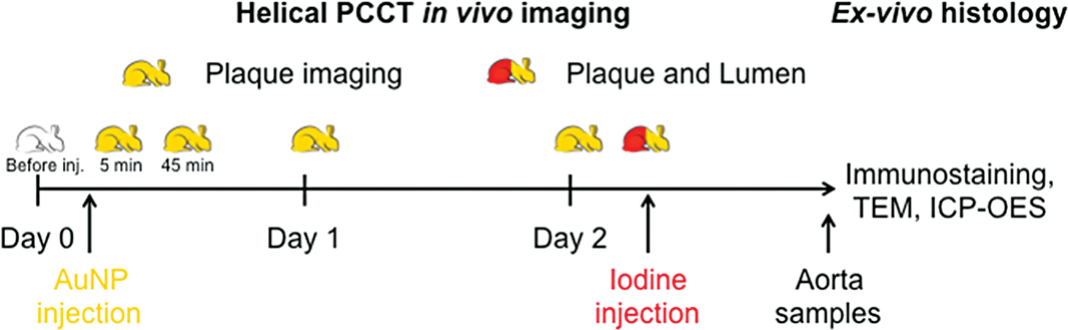
Study design schematic. AuNP = gold nanoparticles, ICP-OES = inductively coupled plasma optical emission spectrometry, inj. = injection, PCCT = photon-counting CT, TEM = transmission electron microscopy.

Helical acquisitions of the whole rabbit aorta were performed using a PCCT system (Philips Healthcare) at 120 kVp and 100 mAs, with a pitch of 1 and a rotation time of 1 second. The PCCT system was a prototype system based on a modified clinical CT system with a field of view of 160 mm and a z-coverage of 2.5 mm. It was equipped with energy-sensitive photon-counting detectors of 2-mm-thick cadmium zinc telluride with a pixel pitch of 270 × 270 µm^2^ at the isocenter. With respect to k-edge imaging, the PCCT system allowed for five energy bins set at 30–53, 53–78, 78–83, 83–98, and 98–120 keV for gold imaging only (ie, monocolor imaging) and gold plus iodine (ie, bicolor imaging) (9,10). In vitro imaging was first performed with tubes containing the k-edge contrast material (ie, gold nanoparticles), an iodinated contrast material, and calcium phosphate. In vivo imaging was performed before and after injection of 3.5 mL/kg of gold nanoparticles (65 mg gold atoms per milliliter of nanoparticles) at 5 minutes, 45 minutes, 1 day, and 2 days. Aortic CT angiography was performed at the end of follow-up with 1 mL/kg intravenous injection of an iodinated vascular contrast material (iomeprol, 400 mg/mL [Iomeron, Bracco]) at a speed of 2 mL/sec to prove the feasibility of bicolor imaging in atherosclerosis. The study design is depicted in Figure 1.

### PCCT Image Reconstruction and Analysis

Conventional images in Hounsfield units and spectral images (ie, gold k-edge, iodine, and water images, scaled in milligrams per milliliter) were reconstructed from the same acquisition using the same reconstruction parameters (voxel size, 250 mm^3^; standard reconstruction kernel). A descriptive qualitative analysis of the aortic wall in terms of presence of hyperattenuated regions before and after injection was performed by two observers in consensus (S.A.S.M., with 10 years of experience, and L.B., with 20 years of experience). For an analysis of the gold nanoparticle uptake within the aortic wall, we performed a semiautomatic quantitative analysis of the macrophage burden by measuring the attenuation on conventional images and the concentration of gold on gold k-edge images. Analysis was performed by segmenting semiautomatically a volume of interest of the whole aortic lumen using dedicated software (Amira, Thermo Fischer Scientific) and a dedicated graphic tablet (Intuos, Wacom). The masks were then transferred to an in-house Matlab routine (Mathworks) to compute a wall mask according to a predefined homogeneous thickness (2 pixels, 500 µm thickness). Furthermore, the total quantity of gold within the segmented wall was calculated according to the mean gold concentration and the total volume of the mask. For an analysis of gold nanoparticle uptake within the immunostained aortic wall sections, we performed a quantitative analysis of five axial sections of 0.25 mm thickness spaced every 5 mm, starting from the first section below the celiac artery (seven rabbits, 35 sections), that corresponded to the slices analyzed at histologic examination. The attenuations on conventional images and gold concentrations on gold k-edge images were measured by segmenting the wall using the freehand selection tool in ImageJ software (National Institutes of Health, *https://imagej.nih.gov/ij/*) on conventional images and transferring the region of interest to the corresponding gold k-edge image (22).

### ex Vivo Analyses

For all atherosclerotic rabbits, five sections of the aorta were collected and analyzed after multiple stainings and imunostaining to score the type of plaque according to the American Heart Association classification (23), to measure the area of macrophages in percentage within the wall, to analyze the distribution of gold nanoparticles with transmission electron microscopy, and to measure the gold content using quantitative inductively coupled plasma optical emission spectrometry.

### Statistical Analysis

Data distributions were tested for normality using the d’Agostino-Pearson test. Data were expressed as means ± standard deviations and ranges in Hounsfield units for conventional images, in milligrams per milliliter for gold k-edge images, and in milligrams for gold quantity measured with inductively coupled plasma optical emission spectrometry and PCCT.

To compare the aortic wall enhancement between time points before injection and 2 days after injection, a two-tailed paired *t* test was performed in the atherosclerotic animal group (*n* = 7) and the control group (*n* = 4).

To assess the correlation between the macrophage area measured in immunostained sections and both the concentration of gold and the attenuation measured in the corresponding PCCT sections (*n* = 35), a two-tailed Pearson correlation coefficient was calculated.

The nominal level of significance for all tests was *P* < .05. Prism software (version 7.0a, GraphPad Software) was used for statistical analysis.

A more detailed description of the methods is given in Appendix E1 (online).

## Results

Seven atherosclerotic New Zealand white rabbits (mean weight, 3.8 kg ± 0.5 [standard deviation]; mean age, 34.5 weeks) and four control nonatherosclerotic New Zealand white rabbits (mean weight, 3.4 kg ± 0.2; mean age, 18 weeks) were included. All rabbits completed the study protocol. PCCT images were available for all animals, with satisfactory image quality at all time points. All data were analyzed.

### in Vitro Characterization and Imaging of the Polyethylene Glycol–coated Gold Nanoparticles

**Figure 2:**
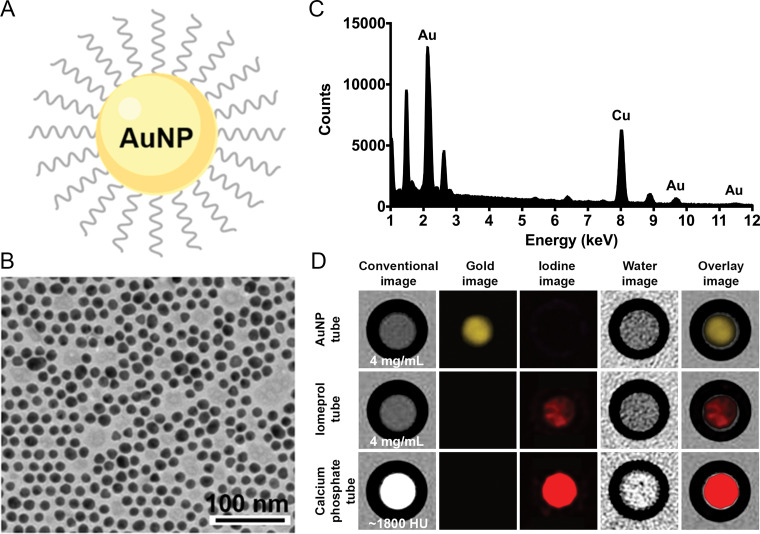
In vitro characterization and imaging of gold nanoparticles (AuNP). *A*, Schematic representation of a gold nanoparticle. *B*, Transmission electron photomicrograph of gold nanoparticle. Note that gold nanoparticles are represented by darker spots. *C*, Characteristic absorption spectrum of gold at energy-dispersive x-ray spectroscopy. Au = gold, Cu = copper. *D*, In vitro photon-counting CT images of tubes containing gold nanoparticles (4 mg/mL), iomeprol (4 mg/mL), or calcium phosphate (1800 HU).

The gold nanoparticles used had a core size of 12.5 nm as determined with transmission electron microscopy and a mean hydrodynamic diameter of 18 nm as determined with dynamic light scattering. Energy-dispersive x-ray spectroscopy resulted in strong peaks at 2.12, 9.66, and 11.50 keV that are characteristic of gold atoms (copper and other peaks are background arising from the grid used). In vitro k-edge imaging depicted only the gold nanoparticle tube, with no background or signal from the iodine and calcium tubes, confirming the capacity to detect specifically gold nanoparticles (Fig 2).

### Macrophage Burden Visualization with Gold K-Edge Imaging in Vivo

**Figure 3:**
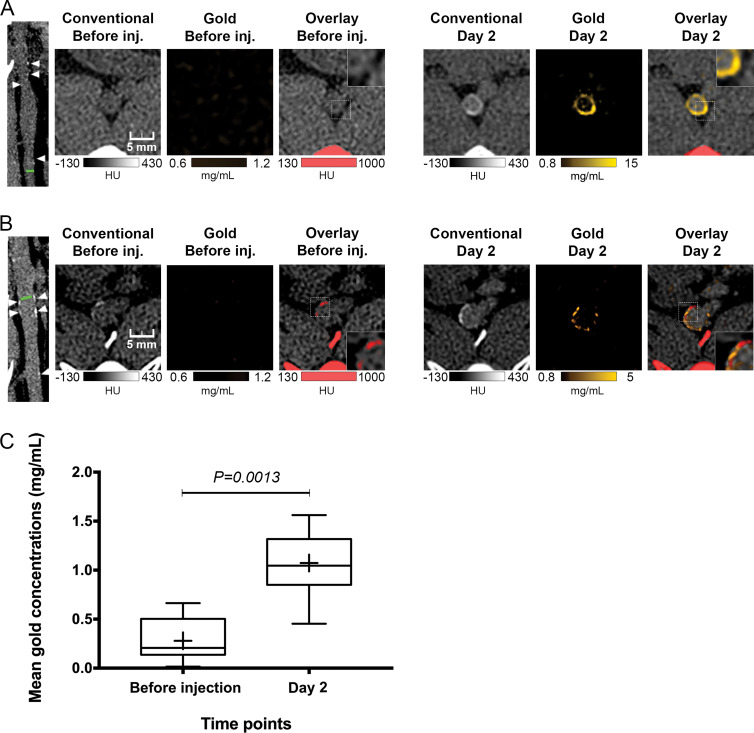
Photon-counting CT images of atherosclerotic rabbit aortas before and 2 days after injection (inj.) of gold nanoparticles. *A*, Images show noncalcified plaque with strong circumferential enhancement and mean wall concentration of 4.5 mg/mL of gold nanoparticles. Parietal calcifications (arrowheads) are also shown. Green line in first image indicates aortic section analyzed. Rectangles in Overlay Before inj. and Overlay Day 2 images represent close-up view of parietal wall for better analyzing enhancement. *B*, Images show calcified plaque with strong enhancement within and around calcified area and mean wall concentration of 2.74 mg/mL of gold nanoparticles. Parietal calcifications (arrowheads) are also shown. Green line in first image indicates aortic section analyzed. Spotty areas of enhancement were more discernable on gold k-edge image than on conventional image. Rectangles in Overlay Before inj. and Overlay Day 2 images represent a close-up view of parietal wall for better analyzing enhancement. *C*, Box-and-whisker plot shows quantitative analysis of mean gold concentration found in whole atherosclerotic rabbit abdominal aortic wall before injection (mean concentration, 0.28 mg/mL ± 0.22; range, 0.01–0.66 mg/mL) and 2 days after injection (mean, 1.07 mg/mL ± 0.36; range, 0.45–1.56 mg/mL). Lower and upper margins of each box indicate 25th and 75th percentiles. Cross represents mean, and line in box represents median. Outliers indicate minimal and maximal values.

Before injection of gold nanoparticles, sections with focal hyperattenuation because of calcifications (Fig 3) on conventional images were observed along the aortic wall in all atherosclerotic animals. The location and extent of these sections were variable among the animals, but they were more predominant within the abdominal aorta, where the diameter was smaller. No hyperattenuation was observed along the aortic wall in nonatherosclerotic animals (Fig E1 [online]).

After injection of gold nanoparticles, plaque enhancement and calcifications were not differentiated on conventional CT images because of similar attenuation values. The detection of any enhancement within a calcification was not possible with conventional images, even when making a comparison between pre- and postinjection images. Uptake of gold nanoparticles was identifiable only on gold k-edge images. Furthermore, enhancement on gold k-edge images was sometimes colocalized with calcifications (Fig 3).

**Figure 4:**
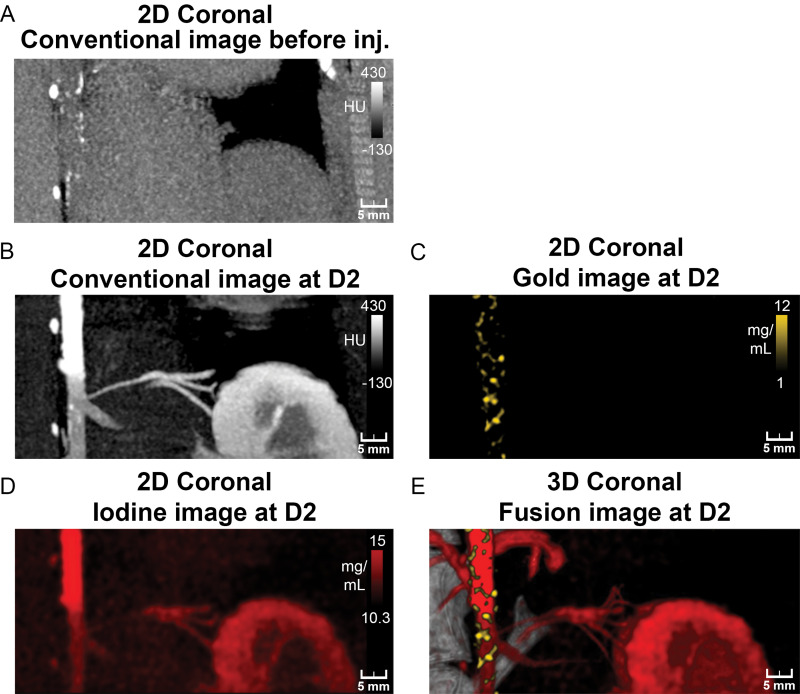
Photon-counting CT images of atherosclerotic rabbit aorta before and 2 days after injection (inj.) of gold nanoparticles. *A*, Coronal 5-mm-width maximum intensity projection conventional image before injection shows presence of focal hyperattenuation along aortic wall indicative of calcifications. *B–D*, Coronal 5-mm-width maximum intensity projection aortic CT angiograms of atherosclerotic rabbit injected with vascular contrast material (iomerprol, 400 mg/mL) at 2 days (D2) after injection of gold nanoparticles. Lumens of aortic and renal arteries and enhancement of kidney and dense wall lesions are apparent on, *B*, maximum intensity projection conventional and, *D*, iodine images. *C*, Coronal 5-mm-width maximum intensity projection gold k-edge image shows extensive heterogeneous enhancement of wall. *E,* Fusion of three-dimensional (3D) volume rendering of conventional images with gold k-edge and iodine images shows spatial distribution of atherosclerotic macrophage burden within aortic wall. 2D = two-dimensional.

**Figure 5:**
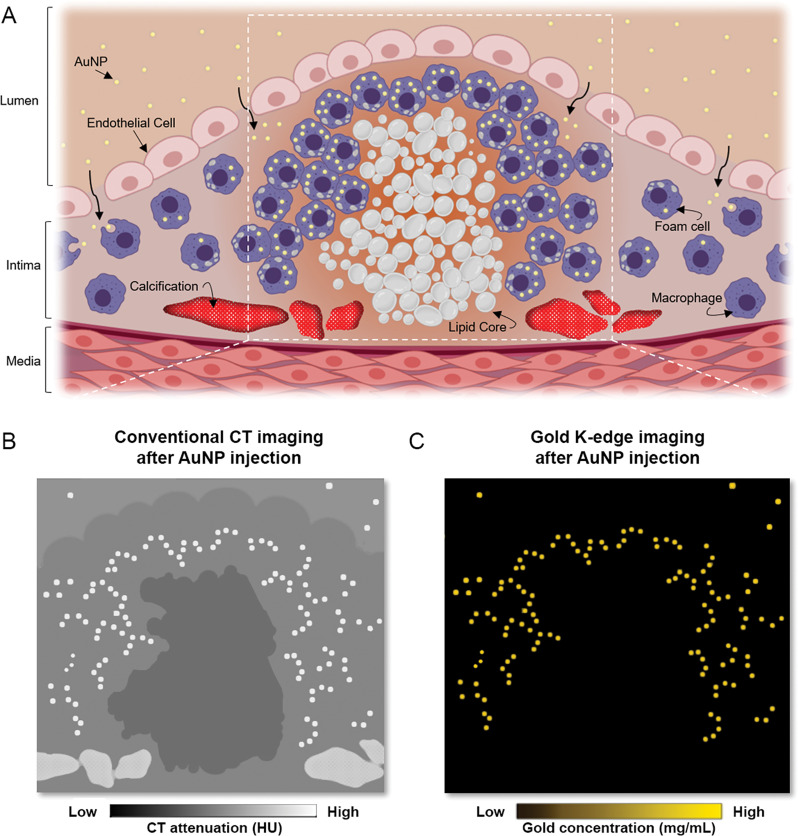
Molecular k-edge imaging approach using photon-counting CT in vivo for macrophage detection and quantification in calcified atherosclerotic plaque in combination with k-edge contrast material of gold nanoparticles (AuNP). *A*, Schematic of uptake of gold nanoparticles by macrophages within atherosclerotic plaque (gold nanoparticles are shown as yellow dots, macrophages and foam cells are shown in purple, lipid core is shown in white, and calcification is shown in red). Gray nodules in foam cells are lipid droplets. White dashed lines indicate field of view of close-up in *B and C*. *B*, Schematic of conventional CT image shows different high-attenuation materials (gold nanoparticles and calcifications) that hamper characterization and quantification of macrophage burden. *C,* Schematic of PCCT gold k-edge image for specific, noninvasive macrophage burden imaging, which permits quantification of gold nanoparticles.

Aortic wall enhancement in atherosclerotic animals was the highest on conventional and gold k-edge images at day 2. At this time point, atherosclerotic aortic walls had extended heterogeneous enhancement in terms of distribution, either large or punctate, and intensity on conventional and gold k-edge images. However, punctate enhancements were more easily detected on gold k-edge images than on conventional images because of the background signal from the wall itself on conventional images. Conversely, no enhancement was observed for control nonatherosclerotic aortas (Figs 3–4, E1–E3 [online]). An illustration of this molecular k-edge imaging approach in combination with gold nanoparticles in calcified atherosclerotic plaque is provided in Figure 5.

At day 2 after injection of an iodinated vascular contrast material, both conventional and iodine images showed good enhancement of the lumen with visualization of an irregular aortic diameter and enlargement of the infrarenal aorta, which indicates aneurismal development, as well as high-attenuating spots within the wall. We did not observe any stenosis, thromboses, or ulcerations in any animal. Conventional and iodine images did not help differentiate between areas of high-attenuating wall and the enhanced lumen. Only gold k-edge images depicted specifically the enhancements within the wall. Visual differentiation between the iodine-enhanced lumen and the gold nanoparticle–enhanced atherosclerotic macrophage burden was thus possible because of the bicolor display of PCCT images (Fig 3).

### Macrophage Burden Quantification in the Aortic Wall with Gold K-Edge Imaging in Vivo

For atherosclerotic rabbits, aortic wall enhancement 2 days after injection of gold nanoparticles on gold k-edge images was higher than that before injection (mean, 1.07 mg/mL ± 0.36 [range, 0.45–1.56 mg/mL] vs 0.28 mg/mL ± 0.22 [range, 0.01–0.66 mg/mL], respectively; *P* = .001) (Fig 3, Table). The same finding was seen with conventional images (mean, 7 HU ± 15 [range, −7 to 33 HU] vs −11 HU ± 21 [range, −35 to 33 HU]; *P* = .003). For control rabbits, no significant increase was measured on gold k-edge images (*P* > .05) and conventional images (*P* > .05).

**Table:**
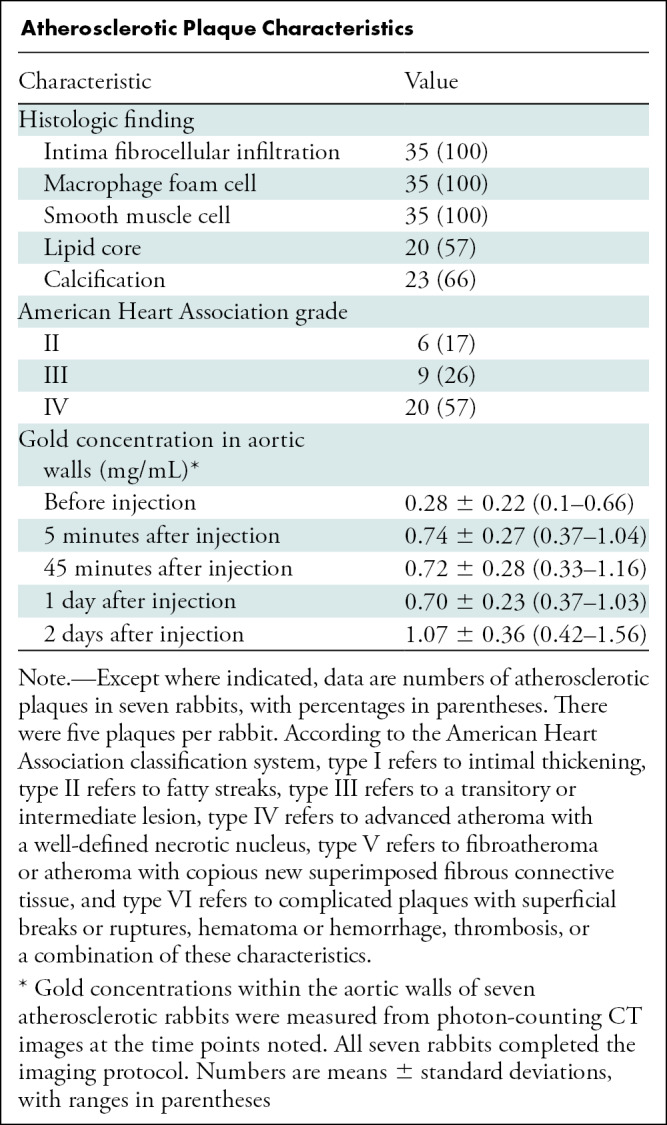
Atherosclerotic Plaque Characteristics

Furthermore, the mean gold concentration on gold k-edge images within the atherosclerotic aortic samples measured with PCCT was 2.73 mg ± 0.92 (range, 1.15–3.98 mg). This high quantity of gold was confirmed with inductively coupled plasma optical emission spectrometry, with a mean concentration of gold measured at 1.53 mg ± 0.55 (range, 0.64–2.17 mg).

### Macrophage Burden Quantification in Atherosclerotic Plaque with Gold K-Edge Imaging in Vivo and Correlation with Macrophage Burden at Immunohistochemistry

Gold concentration was measured 2 days after injection on gold k-edge images in adjacent sections (seven animals and 35 sections analyzed). The mean gold concentration was 2.05 mg/mL ± 1.0 (range, 0.13–4.5 mg/mL), whereas the mean attenuation on conventional images was 70.7 HU ± 23.0 (range, 14.4–138.2 HU). The mean area immunostained for macrophages with RAM11, or monoclonal anti-rabbit macrophage antibody, on corresponding aorta sections was 21.5% ± 10.5 (range, 1.0%–47.1%).

**Figure 6:**
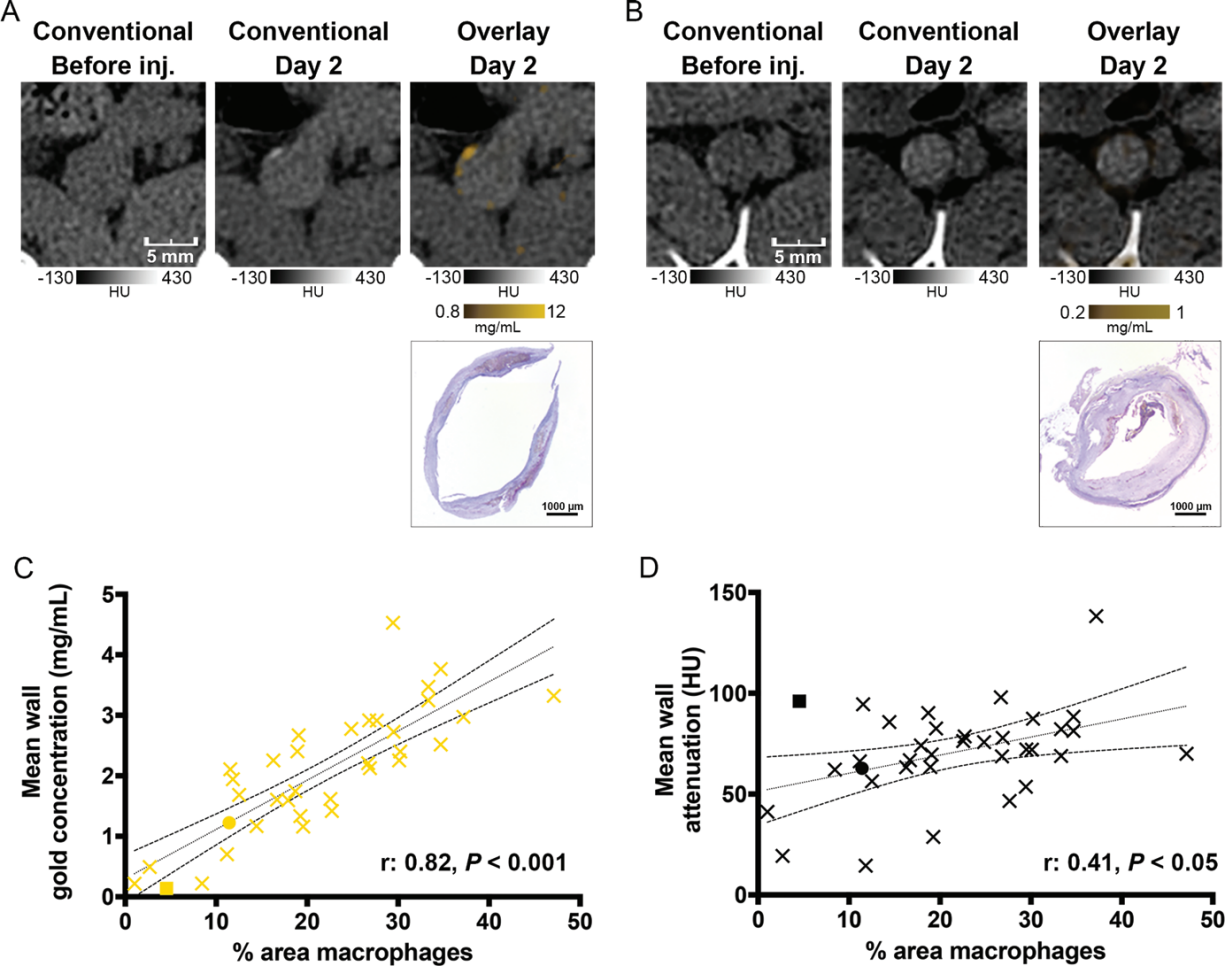
*A*, *B*, Photon-counting CT (PCCT) images of atherosclerotic macrophage burden before injection (inj.) of gold nanoparticles and 2 days after injection and corresponding photomicrographs obtained with immunostaining. *A,* Images of noncalcified plaque show matches of spotty areas of enhancement next to mesenteric artery branch. This section is represented by yellow dot in *C* and black dot in *D*. *B,* Images of calcified plaque show low enhancement on gold k-edge image, which is confirmed by low concentration of immunostained macrophages. This section is represented by yellow square in *C* and black square in *D*. *C, D,* Graphs show correlation between area immunostained for macrophages and, *C,* concentration of gold or, *D,* attenuation on conventional images. Dotted and dashed lines represent the linear regression line and 95% CI.

A good correlation between the gold concentration measured within the wall on gold k-edge images and the macrophage area was found (Pearson correlation: *r* = 0.82; 95% CI: 0.67, 0.91; *P* < .001). Conversely, poor correlation was obtained between the attenuation values measured within the wall on conventional images and the macrophage area (*r* = 0.41; 95% CI: 0.09, 0.65; *P* < .05) (Fig 6).

### ex Vivo Analyses

Twenty of the 35 plaques (57%) were graded as American Heart Association type IV (Table). More importantly, 23 of the 35 plaques (66%) had calcifications. Macrophage foam cells were detected around lipid cores and also in high-infiltrated fibro-inflammatory cells at the intima inner surface, as confirmed by immunostaining with RAM11 (Fig E4 [online]). No macrophage foam cells were found in the media or the adventitia. In nonatherosclerotic rabbits, the lipid core, calcification, and layer disorganization were not observable (Fig E1 [online]).

Transmission electron microscopy confirmed the presence of macrophages and foam cells in high concentrations around lipid cores as well as the presence of gold nanoparticles in the plaques of all atherosclerotic rabbits, although gold nanoparticles were not found in control nonatherosclerotic rabbits. Gold nanoparticles were distributed in high concentrations intracellularly next to lipid inclusions, likely because of phagocytosis by the macrophage-derived cells (Fig E5 [online]). Note that such deposition was not observed neither extracellularly in the extracellular matrix nor intracellularly aside from the foam cells (Fig E6 [online]).

## Discussion

Our results demonstrated specific detection and quantification of macrophage burden in atherosclerotic rabbits using gold k-edge imaging with a photon-counting CT system in combination with gold nanoparticles. We also showed the feasibility of specific differentiation between enhancement of the lumen with one iodinated contrast material and enhancement of the aortic wall with k-edge gold nanoparticles. Our results demonstrated the potential for simultaneous assessment of different diagnostic tasks in atherosclerosis (ie, evaluation of the lumen in terms of stenosis measurement and thrombus detection and evaluation of the plaque in terms of composition and vulnerability).

In vivo macrophage burden CT imaging has previously been reported with an iodinated nanoparticle formulation using conventional CT imaging in atherosclerotic rabbits (24). However, in that study, calcifications in the aortic wall could not be detected. Indeed, materials with different chemical compositions can be represented with similar pixel values on conventional CT images, which means that detection of contrast material uptake in atherosclerotic plaques can be confounded by the presence of calcifications that are frequently observed in coronary artery disease (4,25). When using conventional CT imaging, calcifications will thus interfere with macrophage burden quantification, which is usually observed in early-stage atherosclerosis (26), and subsequently hinder the assessment of vulnerable plaque. Even pre-injection imaging with CT would not be satisfactory because of strong artifacts surrounding the calcifications that could mask small focal enhancement. Therefore, a need exists for a specific imaging technique, such as PCCT k-edge imaging, that, as demonstrated by several proof-of-concept studies, has the capacity to specifically detect and quantify contrast materials (5,9–11,15–17,27,28).

An ideal PCCT contrast material for the evaluation of coronary arteries needs to combine macrophage specificity and a high payload of an element suitable for k-edge imaging, such as ytterbium, gadolinium, or gold nanoparticles, among others (27–29). Previously, Cormode et al (30) reported that using gold nanoparticles in gold k-edge imaging could help detect the macrophage burden in an ex vivo mouse model. However, the animal model and the technical limitations of the system, particularly the low count rate capability and very long scanning time (approximately 12–24 hours), were strong limitations for considering feasibility for coronary artery disease imaging. In our study, using a PCCT prototype system, we demonstrated in vivo that k-edge imaging is a good imaging technique to visualize the macrophage burden even in the presence of calcifications, characterize the plaques by demonstrating the presence of gold nanoparticle uptake in the macrophages, and quantify the macrophage burden with greater accuracy than conventional CT imaging, thus fulfilling the molecular imaging expectations (31). Accordingly, unlike the attenuation of the aortic wall measured on conventional CT images, the quantity of gold nanoparticles in the aortic wall measured on gold k-edge images strongly correlated with the macrophage-covered area at immunostaining. Note that the quantification of a k-edge material has pitfalls as suggested by the nonzero values of gold concentrations before injection of gold nanoparticles. This is likely explained by the noise on the photon counts and physical effects, such as beam hardening, pulse pile-up, and charge sharing, rather than calcium misclassification (32,33). Indeed, calcium has a k-edge effect (4.0 keV) far from the k edge of gold (80.7 keV), such that cross talk is avoided. Moreover, calcium k edge is not measurable because of the absorption of low-energy photons.

Two key factors enable the uptake in macrophages. The first is a long circulation time and penetration into diseased tissue as observed with polyethylene glycol–coated nanoparticles (11,34), and the second is the high concentration of macrophages within atherosclerotic plaques (3,21). In our study, these factors were optimally met 2 days after injection when the specific visualization and quantification of overall macrophage burden at gold k-edge imaging demonstrated the uptake of gold nanoparticles by macrophage foam cells in the aortic wall of atherosclerotic rabbits. This period of time allowed macrophage foam cells to phagocytize enough material to exceed the current sensitivity of k-edge imaging (ie, a concentration in the range of milligrams per milliliter, as suggested before for molecular CT imaging) (9,35). The uptake of the gold nanoparticles by the macrophage foam cells in the aortic wall was also confirmed by macrophage infiltration in corresponding histologic sections, gold nanoparticle uptake by macrophage foam cells at transmission electron microscopy, and gold nanoparticle quantification with inductively coupled plasma optical emission spectrometry in ex vivo aortas.

Another important feature of k-edge imaging is the possibility to perform bicolor imaging, which is mostly performed with optical imaging techniques (31,36). Using this feature, we were able to differentiate the atherosclerotic lumen aorta enlargement labeled with an iodine-based contrast material from the hallmarks of the inflammatory plaques labeled with gold nanoparticles 2 days after injection. Therefore, we confirmed that k-edge imaging presents anatomic information of the lumen and molecular information of the inflammation plaques simultaneously, as suggested in previous studies (10,30), as opposed to alternative imaging modalities such as fluorine 18 fluorodeoxyglucose PET (20). Finally, in addition to k-edge imaging, previous studies have shown that PCCT technology could improve the ability to differentiate atherosclerotic plaque components (eg, lipids and calcification), based on their specific photoelectric and Compton effects (30,37,38), the ability to quantify the vasa vasorum density (ie, a marker of atherosclerotic plaque severity) (39), the visualization of the lumen artery within a stent for diagnosing in-stent restenosis (12,40), and the quantification and measurement reproducibility of coronary Agatson calcium score with a low x-ray radiation dose (14). Taken together, these key features make this modality a promising tool for coronary artery disease imaging and a good candidate for comprehensive identification of adverse plaque features as identified by a recent position paper from the European Society of Cardiology (41).

Our study had several limitations. Comparative investigation of macrophage burden and vascular calcifications that are also considered as a marker of vulnerability within atherosclerotic plaque is lacking (25). The long-term clearance of gold nanoparticles from atherosclerotic plaque was not assessed. Finally, the ability to transfer the study’s findings to humans is unknown because the relationship between gold deposition and plaque rupture was not evaluated.

In conclusion, photon-counting CT–enabled gold k-edge imaging allows for the specific detection and quantification of the macrophage burden within calcified atherosclerotic plaques and for simultaneous anatomic and molecular imaging of atherosclerosis.
